# Novel immunotherapeutic options for BCG‐unresponsive high‐risk non‐muscle‐invasive bladder cancer

**DOI:** 10.1002/cam4.6768

**Published:** 2023-12-01

**Authors:** Zein Alabdin Hannouneh, Amjad Hijazi, Alaa Aldeen Alsaleem, Siwan Hami, Nina Kheyrbek, Fadi Tanous, Karam Khaddour, Abdulfattah Abbas, Zuheir Alshehabi

**Affiliations:** ^1^ Faculty of Medicine Al Andalus University for Medical Sciences Tartus Syrian Arab Republic; ^2^ Cancer Research Center Tishreen University Lattakia Syrian Arab Republic; ^3^ Faculty of Medicine Tishreen University Lattakia Syrian Arab Republic; ^4^ Faculty of Medicine Al‐Baath University Homs Syrian Arab Republic; ^5^ Department of Medical Oncology Dana‐Farber Cancer Institute, Harvard Medical School Boston Massachusetts USA; ^6^ Professor of Nephrology, Faculty of Medicine Al Andalus University for Medical Sciences Tartus Syrian Arab Republic; ^7^ Department of Pathology Tishreen University Hospital Lattakia Syrian Arab Republic

**Keywords:** Bacillus Calmette–Guérin‐unresponsive, gene therapy, immune checkpoint inhibitors, immunotherapy, non‐muscle‐invasive bladder cancer, oncolytic viral therapy, targeted therapy

## Abstract

**Background:**

High‐risk non‐muscle‐invasive bladder cancer (HR‐NMIBC) presents a challenge to many physicians due to its ability to resist Bacillus Calmette–Guérin (BCG) intravesical therapy and the substantial rate of progression into muscle‐invasive bladder cancer (MIBC). Patients who are BCG‐unresponsive have worse prognosis and thus require further management including radical cystectomy (RC), which significantly impacts quality of life. Moreover, the ongoing worldwide shortage of BCG warrants the need for policies that prioritize drug use and utilize alternative treatment strategies. Hence, there is a significant unmet need for bladder preserving therapy in this subset of patients.

**Methods:**

To address this issue, we searched the relevant literature in PUBMED for articles published from 2019 through May of 2023 using appropriate keywords. All clinical trials of patients with HR‐NMIBC treated with immune‐related agents were retrieved from clinicaltrials.gov.

**Findings and Future Perspectives:**

Exploratory treatments for BCG‐Unresponsive HR‐NMIBC included immune checkpoint inhibitors (ICI), oncolytic viral therapy, cytokine agonists, and other immunomodulators targeting TLR, EpCaM, FGFR, MetAP2, and IDO1. Some combination therapies have been found to work synergistically and are preferred therapeutically over monotherapy. Three drugs—pembrolizumab, valrubicin, and most recently, nadofaragene firadenovec‐vncg—have been FDA approved for the treatment of BCG‐unresponsive NMIBC in patients who are ineligible for or decline RC. However, all explored treatment options tend to postpone RC rather than provide long‐term disease control. Additional combination strategies need to be studied to enhance the effects of immunotherapy. Despite the challenges faced in finding effective therapies, many potential treatments are currently under investigation. Addressing the landscape of biomarkers, mechanisms of progression, BCG resistance, and trial design challenges in HR‐NMIBC is essential for the discovery of new targets and the development of effective treatments.

## Graphical abstract

There are limited therapeutic options for BCG‐unresponsive patients with non‐muscle‐invasive bladder cancer. The development of long‐term bladder‐preserving therapies is a significant unmet need for these patients. This article summarizes the current landscape of Immunotherapeutic options beyond immune checkpoint blockers for BCG‐unresponsive, high‐risk non‐muscle‐invasive bladder cancer.

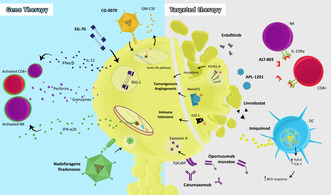

## 1 INTRODUCTION

With an incidence rate of almost half a million per year, bladder cancer (BC) is the ninth most common cancer worldwide.^1^ BC is categorized into muscle‐invasive bladder cancer (MIBC) and non‐muscle‐invasive bladder cancer (NMIBC). It can also be categorized into various subtypes, including micropapillary, squamous and/or glandular, small cell, plasmacytoid, and other subtypes. NMIBC accounts for 75% of BC cases and includes the following pathological stages: noninvasive bladder carcinoma confined to epithelium or mucosa (Ta), tumor invading subepithelial connective tissue (T1), and carcinoma in situ (CIS or Tis).^1^, ^2^ NMIBC is further stratified into three risk groups: low, intermediate, and high risk. The European Organization for Research and Treatment of Cancer (EORTC) established the most reliable risk calculation tables for progression and recurrence based on clinical and pathological factors.^3^, ^4^ High‐risk non‐muscle‐invasive bladder cancer (HR‐NMIBC) includes high‐grade (HG) stage Ta or T1 tumors or any stage with CIS. HR‐NMIBC is a specifically aggressive disease camouflaged under the overall favorable prognosis of NMIBC. This subset of patients is associated with an up to 80% 5‐year risk of recurrence and an up to 50% 5‐year risk of progression, according to EORTC risk tables.^5^ Millan‐Rodriquez et al.^6^ found the rates of recurrence, progression, and mortality in HR‐NMIBC to be 54%, 15%, and 9.5%, respectively. Additionally, tumor characteristics in BCG‐unresponsive NMIBC can be divided into either CIS alone, CIS ± Ta/T1, or papillary only, each of which may harbor variable treatment responses to certain therapies. CIS presents an important prognostic factor when found concomitantly with T1HG tumors. Concomitant CIS in patients with T1HG tumors raise the probability of progression at 1 and 5 years from 10% and 29% to 29% and 74%, respectively.^5^ EORTC evaluated the estimated 1‐ and 5‐year disease‐specific death rates of T1 HG tumors to be 4.8% and 11.3%, respectively.^3^ Likewise, different subtype histologies are also associated with worse oncological outcomes. Micropapillary, plasmacytoid, and small cell subtypes were found to be independently associated with increased risk of overall mortality.^7^ As a result, subtype histology should be considered in the prognosis of BC and in guiding risk stratification.^8^

The initial treatment for intermediate and HR‐NMIBC involves transurethral resection of the bladder tumor (TURBT) followed by intravesical Bacillus Calmette–Guérin (BCG), which has been the standard of care for decades.^9^ However, ongoing BCG shortages in many countries raise concerns and highlights the need for effective alternative therapies and drug prioritization.^10^ Therefore, intermediate‐risk patients are sometimes treated with TURBT followed by alternative intravesical chemotherapy, while BCG is reserved for high‐risk populations.^9^ Besides BCG shortages, BCG has a moderate toxicity profile; most adverse events (AE) are not severe (<5%) and are predominantly due to improper intravesical instillation leading to systemic absorption.^11^ Several randomized controlled trials (RCTs) have shown that intravesical BCG therapy that includes an induction course of 6 weekly instillations and a maintenance course every 3–6 months for over 1–3 years is superior to TURBT alone^12^, ^13^, ^14^, ^15^, ^16^ or TURBT followed by intravesical induction chemotherapy^15^, ^17^, ^18^ in terms of recurrence and progression prevention in NMIBC patients. However, recent studies are demonstrating promising outcomes with the combination of two chemotherapeutic agents (gemcitabine and docetaxel) in the treatment of HR‐NMIBC.^19^, ^20^ In a retrospective review, McElree et al.^21^ found 84% 2‐year high‐grade recurrence free survival (HG‐RFS) using gemcitabine and docetaxel for BCG‐naïve HR‐NMIBC. Further long‐term, retrospective analysis in a BCG‐unresponsive cohort found a 75% 5‐year bladder preservation rate, a 91% 5‐year cancer‐specific survival (CSS) rate, a 75% cystectomy free survival and, a 30% 5‐year HG‐RFS. However, additional prospective trials are imperative.^22^ Nonetheless, induction BCG instillations, given once weekly for 6 weeks, remain the standard of care. Although there can be differences in dosage and duration, additional BCG maintenance therapy further decreases recurrence rates when compared to induction therapy alone.^23^ As a result, a maintenance course is required in patients with HR‐NMIBC. The United States Food and Drug Administration (FDA) defined adequate BCG therapy as at least 5 out of 6 induction doses and 2 out of 3 maintenance doses.^24^

Despite BCG therapy, a significant portion of patients do not respond or experience recurrence, developing BCG failure. Approximately 20% of HR‐NMIBC patients progress into MIBC despite BCG treatment.^25^ In the long term, BCG failure can occur in as many as 50% of patients.^26^ BCG failure definitions can vary depending on different subgroups. Patients who are “BCG intolerant” are those who cannot receive BCG treatment due to adverse effects. Patients with HG cancer 6 months after induction therapy or cancers that have progressed by stage or grade fall under the “BCG refractory” subgroup. “BCG relapsing” subgroup comprises patients with recurrent cancer after a disease‐free state of 6 months after treatment. Due to diverse definitions of BCG failure, the International Bladder Cancer Group (IBCG) and the Genitourinary American Society of Clinical Oncology Group adopted the term “BCG Unresponsive,” which includes both BCG‐refractory and early BCG‐relapsing disease within 6 months.^27^ According to the FDA, BCG unresponsiveness is defined as HG disease following BCG induction course, recurrent HG Ta/T1 within 6 months of adequate BCG therapy, or persistent or recurrent CIS alone or with Ta/T1 within 12 months of adequate BCG therapy.^24^ The consensus on definitions of BCG failure allows for a better understanding and framework of clinical trials.

BCG failure infers poor prognosis. Patients who do not achieve a complete response (CR) post BCG induction have a reduced 5‐year survival rate and an increased risk of a worsening event (cystectomy, chemotherapy, radiotherapy, progression, or death).^28^ Additional BCG therapy after failure is ineffective, and radical cystectomy (RC) remains the standard of care for BCG‐unresponsive patients.^9^, ^29^ RC involves permanent distortion of body image and several functional complications. Furthermore, RC with subsequent urinary diversion is a difficult surgery that is associated with significant perioperative patient mortality (1%–3%),^30^ which can reach 8% when the disease is muscle‐invasive.^31^ Approximately 60% of patients experience early complications, and surgery‐related morbidity is a major concern.^32^, ^33^ As a result, surgery becomes inapplicable for many patients, and many choose to electively decline surgery. Therefore, the development of non‐surgical treatment options for BCG‐unresponsive NMIBC is a priority to improve patient outcomes and lifestyle. Several studies have shown no significant differences in long‐term survival outcomes, including 10‐year overall survival (OS) and CSS when comparing bladder preserving therapies (chemotherapy/immunotherapy) before RC to an early RC.^34^ These findings, in addition to patients' choice or ineligibility for an RC, prompted the investigation of several novel agents in the setting of BCG failure. Clinical trials have evaluated intravesical chemotherapeutic regimens including mitomycin C,^35^, ^36^, ^37^, ^38^, ^39^ gemcitabine,^39^, ^40^, ^41^, ^42^, ^43^, ^44^, ^45^ docetaxel,^43^, ^44^ everolimus,^45^ and valrubicin,^46^ of which only valrubicin attained approval for BCG‐refractory HR‐NMIBC.^47^ There is increased heterogeneity in studies involving chemotherapeutic agents for BCG‐unresponsive disease.^48^ Hence, arriving at definitive oncological outcomes is challenging despite some promising results.

As of July 2023, three agents, valrubicin, pembrolizumab, and nadofaragene firadenovec‐vcng, have been FDA approved in BCG‐unresponsive patients who refuse or are ineligible for RC.^47^, ^49^, ^50^ Valrubicin demonstrated a promising CR of 21% in patients with BCG‐ refractory CIS^46^ which led to its FDA approval in 1998.^47^ However, most studies prior to 2018 had heterogeneity in patient populations and endpoints as there was no established definition for BCG‐unresponsiveness. The FDA published official clinical trial guidance in 2018 that provided a standardized definition of BCG‐Unresponsive patients.^24^ However, the heterogenous nature of NMIBC remains a challenge for clinical trial design. Additionally, the scarcity of RCTs in BCG‐unresponsive disease limits the capacity to clinically adopt new agents. Despite the demonstrated efficacies of pembrolizumab and nadofaragene firadenovec‐vcng, the unavailability of long follow‐up durations creates a gap in our knowledge of long‐term disease control. Treatment‐related adverse events (TRAE), immune‐related adverse events (IRAE), and cost‐effectiveness are additional challenges that need to be addressed in immunomodulatory agents like pembrolizumab. Current alternative agents to RC in HR‐NMIBC (valrubicin, pembrolizumab, and nadofaragene firadenovec‐vncg) tend to postpone progression rather than achieve long‐term disease control. Many clinical trials are still ongoing, and effective alternatives to RC in BCG‐unresponsive patients remain an important unmet clinical need. This review aims to outline the current landscape of novel immunotherapeutic bladder‐preserving therapies, including immune checkpoint inhibitors (ICI), oncolytic viral therapy, cytokine agonists, and other immunomodulators, as well as the utilization of combination therapy for BCG‐unresponsive HR‐NMIBC (Tables 1 and 2).

**TABLE 1:** Clinical trials evaluating immune checkpoint inhibitors (in bold) in high‐risk non‐muscle‐invasive bladder cancer.

NCT/Acronym	Status	Phase	Population	Est. enroll.	Intervention(s)	MOA/Type	Primary end points	Year started
NCT02324582^206^, ^210^	Unknown	1	BCG‐recurrent/presistant	13	**Pembrolizumab** + BCG	Anti‐PD‐1	AE	2015
NCT02625961 KEYNOTE‐057^70^	Recruiting	2	BCG‐unresponsive	320	**Pembrolizumab** vs. **Pembrolizumab** + **Vibostolimab** or **Favezelimab**	Anti‐PD‐1, Anti‐TIGIT, Anti‐ LAG‐3	CRR, DFS	2016
NCT02808143^207^	Active, Not recruiting	1	BCG‐recurrent	9	**Pembrolizumab** + BCG	Anti‐PD‐1	MTD	2017
NCT03504163^73^	Recruiting	2	BCG‐naive	37	**Pembrolizumab** + BCG		DFS	2018
NCT03711032 KEYNOTE‐676^74^	Recruiting	3	BCG naive/BCG‐recurrent/persistent	1405	**Pembrolizumab** + BCG		CRR EFS	2018
NCT04164082^171^	Recruiting	2	BCG‐unresponsive	161	**Pembrolizumab** + Gemcitabine		CRR EFS	2020
NCT04387461 CORE‐001^112^	Active, Not recruiting	2	BCG‐unresponsive	35	**Pembrolizumab** + CG0070		CRR	2020
NCT05843448^178^	Recruiting	1	BCG‐unresponsive/intolerant	30	**PD‐L1/IDO peptide vaccine** + **pembrolizumab IV**		AE	2023
NCT03317158 ADAPT‐BLADDER^83^	Recruiting	1 2	BCG‐unresponsive, BCG‐relapsing, BCG‐naïve	55	**Durvalumab** vs. **Durvalumab** + BCG or Radiotherapy or Gemcitabine/Docetaxel or **Tremelimumab**	Anti‐PD‐L1, Anti‐CTLA‐4	RP2D CRR	2018
NCT03528694^84^	Recruiting	3	BCG‐naive	1018	**Durvalumab** + BCG vs. BCG	Anti‐PD‐L1	DFS	2018
NCT03258593^86^	Completed	1	BCG‐unresponsive	15	**Durvalumab** + Vicinium (oportuzumab monatox)		AE	2018
NCT03759496^85^	Active, Not recruiting	2	BCG‐unresponsive/intolerant/refractory	39	**Durvalumab**		MTD HGFR	2018
NCT05120622^87^	Recruiting	1 2	BCG‐unresponsive	48	**Tremelimumab** and **Durvalumab**	Anti‐CTLA‐4, Anti‐PD‐L1	Grade 3 AE, MTD	2021
NCT02792192^76^	Terminated	1 2	BCG‐unresponsive, relapsing, and naive	24	**Atezolizumab** vs. **Atezolizumab** + BCG	Anti‐PD‐L1	MAD, DLT, AE, CRR	2016
NCT03799835 ALBAN^78^	Recruiting	3	BCG‐naive	516	BCG vs. **Atezolizumab** + BCG		RFS	2019
NCT04134000 BladderGATE^77^	Active not recruting	1	BCG‐naive	40	**Atezolizumab** + BCG		DLT	2020
NCT03892642 ABC Trial^209^	Active, Not recruiting	1 2	BCG‐unresponsive	18	**Avelumab** + BCG	Anti‐PD‐L1	DLT	2019
NCT03950362 PREVERT^208^	Not yet recruiting	2	BCG‐unresponsive	67	**Avelumab** + Radiotherapy		High‐risk RFS	2020
NCT04922047 TACBIN‐01^186^	Recruiting	1 2	BCG‐naive	6	**Tislelizumab** + BCG	Anti‐PD‐1	DLT	2021
NCT05495724 TRUCE‐04^188^	Recruiting	2	Her2 overexpressing	176	**Tislelizumab** + Disitamab Vedotin		CRR	2021
NCT05418309 TRUCE‐22^174^	Recruiting	2	Not completely resectable HR‐NMIBC	63	**Tislelizumab** + Nab‐Paclitaxel		CRR	2021
NCT05580354 SPARE‐007^187^	Not Yet Recruiting	4	BCG‐naive	42	**Tislelizumab** + BCG		RFS	2022
NCT03519256 CheckMate 9UT^168^	Terminated (insufficient enrollment)	2	BCG‐Unresponsive	142	**Nivolumab** vs. **Nivolumab** + BCG or BMS‐986205 (Linrodostat) vs. **Nivolumab** + BCG + BMS‐986205	Anti‐PD‐1	AE, IMAE, SAE	2018
NCT04149574 CheckMate 7G8^80^	Active, not recruiting	3	BCG persistent/recurrent disease ≤24 months of last BCG dose	13	**Nivolumab** + BCG vs. BCG		EFS	2020
NCT04640623 SunRISe‐1^172^	Recruiting	2	BCG‐unresponsive	200	TAR‐200 vs. **Cetrelimab** vs. **Cetrelimab** and TAR‐200	Anti‐PD‐1	CRR	2020
NCT05714202 SunRISe‐3^171^	Recruiting	3	BCG‐naive	1050	TAR‐200 vs. **Cetrelimab** vs. **Cetrelimab** and TAR‐200		EFS	2023
NCT04165317 CREST^89^	Active, Not recruiting	3	BCG‐naive, BCG‐unresponsive	1070	**Sasanlimab PF‐06801591** vs. Sasanlimab + BCG vs. BCG	Anti‐PD‐1	CRR EFS	2019
NCT04738630^184^	Recruiting	2	BCG‐unresponsive	110	**Pucotenlimab (HX008)**	Anti‐PD‐1	CRR EFS	2020
NCT04706598^185^	Recruiting	1 2	BCG‐unresponsive	56	**Camrelizumab**	Anti‐PD‐1	MTD HGFR	2021

Abbreviations: AE, adverse events; BCG, Bacillus Calmette–Guérin; CRR, complete response rate; CTLA‐4, cytotoxic T‐lymphocyte antigen 4; DFS, disease‐free survival; DLT, dose‐limiting toxicity; EFS, event‐free survival; HGFR, high‐grade free recurrence; IMAE, Immune‐mediated adverse events; LAG‐3, lymphocyte‐activation gene 3; MAD, maximum administered dose; MTD, maximum tolerated dose; PD‐1, programmed cell death‐1; PD‐L1, programmed cell death ligand 1; RP2D, recommended phase 2 dose; SAE, serious adverse events; TIGIT, T‐cell immunoglobulin and immunoreceptor tyrosine‐based inhibitory motif domain.

**TABLE 2:** Clinical trials evaluating other agents (in bold) in high‐risk non‐muscle‐invasive bladder cancer.

NCT/Acronym	Status	Phase	Population	Est. enroll.	Intervention	MOA/Type	Primary End points	Year started
**Targeted therapy**								
NCT00330707^204^	Completed	2 3	BCG‐naive	140	BCG vs. **IFN‐α** + BCG	Interferon	Local and systemic toxicity, RR, PR, DST	1995
NCT01118351^211^	Terminated	2	BCG‐refractory	19	**Sunitinib**	RTK inhibitor	CRR	2008
NCT00794950^182^	Completed	2	BCG‐naive	43	BCG + **Sunitinib**		CRR	2009
NCT01732107 GU12‐157^159^	Terminated	2	BCG‐refractory, FGFR3 overexpression or FGFR3 mutation	13	**Dovitinib**	RTK inhibitor (FGFR + PDGFR)	CRR	2013
NCT04498702^161^	Completed	2	BCG/Chemo‐relapsed	41	**APL‐1202**	MetAP2 inhibitor	RFR	2014
NCT04490993^161^	Active, not recruiting	3	Chemo‐refractory	359	**APL‐1202** + Epirubicin vs. Epirubicin		EFS	2017
NCT02449239^153^	Unknown	3	BCG‐refractory	133	**Vicinium (oportuzumab monatox)**	EpCaM binding	CRR	2015
NCT03022825^126^	Recruiting	2 3	BCG‐unresponsive	190	BCG + **N‐803**	IL‐15 superagonist	CRR, DFS	2017
NCT04172675^39^	Active, not recruiting	2	BCG‐recurrent, FGFR mutations or fusions	105	**Erdafitinib** vs. MMC or Gemcitabine	FGFR kinase inhibitor	RFS	2020
NCT05410730^196^	Recruiting	1 2	BCG‐naive	105	BCG vs. BCG + **SHR‐1501**	IL‐15 superagonist	DLT, MTD, RP2D, CRR, DFS	2022
NCT05710848^143^	Recruiting	1 2	Recurrent after SOC	75	**STM‐416 (Resiquimod)**	TLR7,8 agonist	RFS, AE, DLT	2023
NCT05014139 EV‐104^199^	Recruiting	1	BCG‐unresponsive	58	**Enfortumab vedotin**	Anti‐Nectin‐4	AE, DLT, ILA	2021
NCT05768347^205^	Recruiting	1	BCG‐exposed	12	**Adoptive Cell Therapy**	Tumor‐infiltrating lymphocytes	AE	2023
**Gene therapy**								
NCT00406068^201^	Completed	2 3	BCG‐refractory	129	**MCNA**	Immune and apoptotic mechanism	DFS	2006
NCT02365818^109^	Completed	2	BCG‐unresponsive	66	**CG0070**	Ad5 oncolytic w/ E2F promotor and GM‐CSF	DCR	2015
NCT04452591^111^	Recruiting	3	BCG‐unresponsive	110	**CG0070**		CRR	2020
NCT01687244^99^	Completed	2	BCG‐refractory	40	INSTILADRIN (**nadofaragene firadenovec‐vcng**)	Ad vector w/ IFNα‐2b	RFS	2012
NCT02773849^100^	Completed	3	BCG‐unresponsive	157	ADSTILADRIN (**nadofaragene firadenovec‐vcng**)		CRR	2016
NCT05704244^101^	Recruiting	3	BCG‐unresponsive	24	FE 999326 (**nadofaragene firadenovec‐vcng**)		CRR	2022
NCT04752722^120^	Recruiting	1 2	BCG‐unresponsive, BCG naive, incomplete BCG treatment	222	**EG‐70**	Encoding two RIG‐1 agonists and IL‐12	AE and SAE, CRR	2021
NCT05085977 ADVANCED‐1^144^	Recruiting	1a	BCG‐intolerant, BCG‐naive (24 m), BCG‐recurrent/persistent	18	**TARA‐002**	Immunopotentiator, modified group A streptococcus pyogenes	DLTs, AE, RP2D	2022
NCT05085990 ADVANCED‐1^144^	Recruiting	1b		12			AE	2023
NCT05951179 ADVANCED‐2^145^	Not yet recruiting	1 2		102			High‐grade CRR	2023

*Note*: Green‐shaded region belong to targeted therapy and the blue‐shaded region belong to gene therapy.

Abbreviations: Ad, adenovirus; AE, adverse events; BCG, Bacillus Calmette–Guérin; CRR, complete response rate; DCR, durable complete response; DFS, disease‐free survival; DLT, disease‐limiting toxicity; DST, disease‐specific toxicity; EFS, event‐free survival; FGFR, fibroblast growth factor receptor; GM‐CSF, granulocyte‐macrophage colony stimulating factor; ILA, incidence of laboratory abnormalities; m, months; MCNA, mycobacterium phlei cell wall‐nucleic acid complex; MTD, maximum tolerated dose; PDGFR, Platelet‐derived growth factor receptor; PR, progression rate; RFR, recurrence free response; RFS, recurrence free survival; RIG‐1, retinoic acid‐inducible gene‐1; RP2D, recommended phase two dose; RR, recurrence rate; RTK, receptor tyrosine kinase; SAE, serious adverse events; TLR, toll‐like receptor.

## 2 BCG STRAINS

An increased global demand for BCG in the presence of decreased production by pharmaceutical companies gave rise to BCG shortages across the globe.^51^ Merck, the sole provider of worldwide TICE BCG, is expected to raise its supply by 2026.^52^ This shortage is still ongoing and is expected to continue for years. The development of alternative BCG strains with comparable efficacy is a possible solution. Currently, only TICE strain is predominantly used in the US, while Armand‐Frappier and Connaught are not currently in production but are FDA‐approved and can interchangeably lead to similar outcomes.^53^, ^54^ Many studies have evaluated the difference in oncological outcomes, such as recurrence‐free survival (RFS) and progression‐free survival (PFS), between BCG strains in NMIBC. Tokyo‐172, Pasteur, and TICE strains demonstrated no significant superiority in terms of BC recurrence rates over each other in a network meta‐analysis.^55^ Moreover, BCG‐Moreau and BCG‐Tice showed no difference in RFS or PFS in a retrospective study of patients with NMIBC cases.^56^ A higher incidence of AE was reported in the Moscow strain when compared to the Danish strain, but there was no difference in RFS or PFS.^57^ No difference was observed in BCG RIVM and Tice in terms of PFS and CSS in a retrospective study, though an increased RFS was outlined in Tice and RIVM.^58^ Overall, the development of new, accessible strains of BCG may address the issue of BCG shortages, especially when most strains of BCG have shown comparable oncological outcomes.

## 3 IMMUNE CHECKPOINTS IN BLADDER CANCER

Programmed cell death‐1 (PD‐1) is a membrane receptor expressed on activated T‐cells that interacts with its ligands, programmed cell death ligand 1 and 2 (PDL‐1, PDL‐2) to predominantly suppress T‐cells in peripheral tissues and lymphoid organs, respectively.^59^ PD‐L1 and PD‐L2 differ in their molecular mechanisms of interaction with PD‐1. PD‐L1 is predominantly expressed on a broad spectrum of non‐hematopoietic and hematopoietic cells, including T and B‐cells, macrophages and DCs, whereas PD‐L2 expression is typically reserved for activated B‐cells, DCs, and monocytes.^59^ Cytotoxic T‐lymphocyte antigen 4 (CTLA‐4) is another immune checkpoint that acts as a negative regulator of T‐cell response.^60^ These factors assist in maintaining immune homeostasis and safeguarding against autoimmunity; however, using PD‐1, PD‐L1, or CTLA‐4 monoclonal antibodies can be useful in upregulating T‐cell response against tumor cells.

The discovery of ICI, such as anti‐PD‐1, anti‐PD‐L1, and anti‐CTLA4, allowed the development of treatments that can significantly generate antitumor activity, especially in tumors with high tumor mutational burden (TMB). Urothelial bladder carcinoma has a higher TMB than that of many other common cancers, which is close to that of melanoma and lung cancer.^61^ The high mutational rate allows for the increased release of neoantigens, which in turn enhances the effects of ICI.^61^ As a result, many ICI, including atezolizumab, nivolumab, durvalumab, and pembrolizumab, are currently the classic second‐line treatments for MIBC and advanced urethral carcinomas.^62^, ^63^, ^64^, ^65^ Subsequently, the use of ICI is now being investigated in NMIBC, especially in the setting of BCG failure, where drug choice is limited and RC is optimal.

### 3.1 Immune checkpoint inhibitors in BCG‐unresponsive NMIBC

**FIGURE 1:**
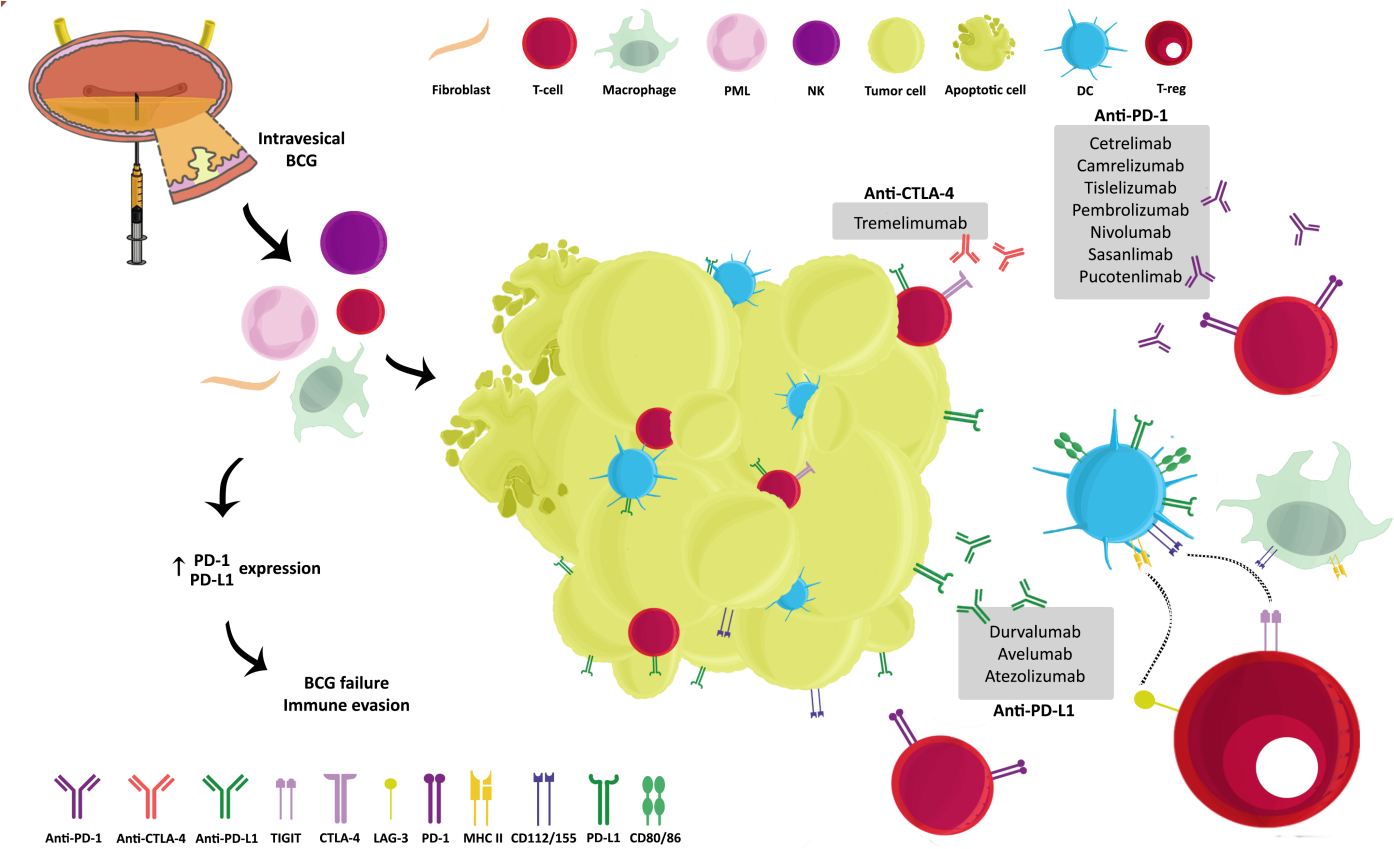
Interplay between Bacillus Calmette–Guérin (BCG) and immune‐checkpoint inhibitors in non‐muscle‐invasive bladder cancer. BCG acts through innate and adaptive immune responses to induce anti‐tumor activity. Programmed cell death‐1 (PD‐1) and cytotoxic T‐lymphocyte antigen 4 (CTLA‐4) interact with their ligands programmed cell death‐ ligand 1 PD‐L1 and CD80/88, respectively, to predominantly suppress T‐cell response.^59^, ^60^ Patients with increased PD‐1 and PD‐L1 expression in the tumor microenvironment tend to be BCG‐unresponsive due to enhanced tumor immune evasion.^67^, ^68^ Immune checkpoint inhibitors counteract immune escape mechanisms of bladder tumor cells by targeting PD‐1, PD‐L1, and CTLA‐4. Anti‐PD‐1, anti‐PD‐L1, and anti‐CTLA‐4 agents that have been used in clinical trials for high‐risk non‐muscle‐invasive bladder cancer are written in boxes. Lymphocyte‐activation gene 3 (LAG‐3),^194^ which binds to major histocompatibility complex class II (MHC ll),^189^ and T‐cell immunoglobulin and immunoreceptor tyrosine‐based inhibitory motif domain (TIGIT),^192^, ^193^, ^194^ which binds to binds to CD112/155,^191^ are other suggested immune checkpoints that are yet to show results in clinical trials involving this subset of patients. BCG, Bacillus Calmette–Guérin; CTLA‐4, cytotoxic T‐lymphocyte antigen 4; DC, dendritic cells; LAG‐3, lymphocyte‐activation gene 3; MHC II, major histocompatibility complex class II; NK, natural killers; PD‐1, programmed cell death‐1; PD‐L1, programmed cell death ligand 1; PML, polymorphonuclear leukocytes; TIGIT, T‐cell immunoglobulin and immunoreceptor tyrosine‐based inhibitory motif domain.

A number of studies have demonstrated that PD‐1 and its ligand, PD‐L1, may play a role in the mechanism of BCG resistance. In a cohort of 63 NMIBC cases, patients who were unresponsive to BCG had an increased expression of PD‐L1+ cells, whereas PD‐L1+ cells were almost absent in BCG responders.^66^ PD‐1 expression was also dominant in BCG‐unresponsive tumors.^67^ Moreover, BCG therapy can enhance PD‐L1, but not PD‐L2, expression on tumor cells and inflammatory T‐cells in tumoral and non‐tumoral tissue, which may explain the immune escape mechanism observed in BCG‐unresponsive cases (Figure 1).^67^, ^68^ Furthermore, enhanced PD‐L1 expression before therapy was associated with unfavorable clinical and pathological outcomes.^68^, ^69^ PD‐L1 positivity is desirable in the context of treatment targeting the PD‐1–PD‐L1 interaction, as the absence of this targeted therapy benefits tumor cells through immune evasion. As a result, both PD‐1 and PD‐L1 may be used as markers for BCG response. These findings provided the groundwork for several clinical trials evaluating the efficacy of ICIs as monotherapy or in combination in HR‐NMIBC (Table 1) (Figure 1).

#### 3.1.1 Pembrolizumab

In an ongoing randomized clinical trial KEYNOTE‐057, Pembrolizumab, a PD‐1 inhibitor, has shown promising results in the treatment of high‐risk BCG‐unresponsive NMIBC, which led to its recent FDA approval.^49^, ^70^, ^71^ With a favorable safety profile and a median follow‐up of 36.4 months in cohort A,^70^ which included BCG‐unresponsive CIS with or without papillary tumors, 39 (41%) out of 96 had a CR at 3 months. Serious TRAEs occurred only in eight (8%) patients. Follow‐up results revealed that 33.3% and 23.1% of the complete responders maintained their response for longer than 18 and 24 months, respectively. As a result, better alternatives may be required for long‐term disease control. Cohort B^71^ included BCG‐unresponsive papillary HR‐NMIBC without CIS and demonstrated HR‐NMIBC disease‐free survival (DFS) of 43.5% and any‐disease DFS of 41.7%. TRAEs occurred in 63% and 73% of cohorts A and B, respectively.^70^, ^71^ 10.6% of patients discontinued treatment due to a TRAE in cohort B.^71^ IRAEs are additionally a concern with pembrolizumab. IRAEs occurred in 22% of patients in cohort A.^70^ Moreover, pembrolizumab remains an expensive alternative, demonstrating no cost‐effectiveness in BCG‐unresponsive patients when compared to RC or salvage intravesical chemotherapy.^72^

In another non‐randomized, Phase II trial (NCT03504163),^73^ pembrolizumab and BCG are being tested as first‐line treatment of HG, BCG‐naive NMIBC. The primary endpoint is the proportion of disease‐free patients throughout 6 months, and results are still awaited. Compared to BCG alone, pembrolizumab and BCG are also being evaluated in a randomized, Phase III trial KEYNOTE‐676^74^ in BCG‐naive and BCG‐recurrent or persistent patients. Results are expected in 2028. The efficacy of combinational treatment (pembrolizumab and BCG) is yet to be established.

#### 3.1.2 Atezolizumab

Atezolizumab is a monoclonal antibody that inhibits the PD‐L1/PD‐1 pathway and blocks CD80 receptors.^75^ In a non‐randomized, Phase Ib/II study (NCT02792192),^76^ 24 participants with BCG‐unresponsive NMIBC were enrolled. 33.3% and 41.7% of patients receiving atezolizumab alone and atezolizumab plus BCG, respectively, had a CR at 6 months of follow‐up.^76^ Other assessed primary outcomes were dose‐limiting toxicity (DLT), maximum administered dose (MAD), and AE.

In another Phase Ib study BladderGATE^77^ enrolled 40 patients with BCG‐naïve HR‐NMIBC. Atezolizumab plus BCG were evaluated with a longer follow‐up period of 24 months to evaluate DLT and RFS. In the latest Phase III study ALBAN,^78^ 516 patients will be randomized in 45 centers in Europe corresponding to a 1:1 ratio, in arm A (BCG control) and arm B (experimental BCG plus atezolizumab).

#### 3.1.3 Nivolumab

Nivolumab, a PD‐1 inhibitor, has been recently approved by the FDA as adjuvant second‐line treatment for metastatic BC,^63^, ^79^ which paved the way for its evaluation as a novel treatment for BCG‐unresponsive HR‐NMIBC.

CheckMate 7G8 (NCT04149574)^80^ is a Phase 3, randomized trial investigating nivolumab in combination with BCG compared to BCG alone in patients with persistent or recurrent high‐risk NMIBC after one adequate course of BCG induction only. The primary outcome is event‐free survival (EFS).

#### 3.1.4 Durvalumab

Durvalumab is a human monoclonal IgG kappa antibody against PD‐L1 that does not induce antibody‐dependent cellular cytotoxicity.^81^ It has been approved by the FDA for advanced or metastatic biliary tract cancer and is being evaluated in ongoing trials for its efficacy in NMIBC patients.^82^

Durvalumab was found to be safe in combination with BCG or external beam radiation therapy (EBRT) in a Phase I trial (NCT03317158)^83^ where 12‐month CR was achieved in 46% of all patients, 73% of durvalumab plus BCG, and 33% of durvalumab plus EBRT arms. The recommended Phase 2 dose (RP2D) for each regimen was the primary endpoint. A Phase III trial (NCT03528694)^84^ assessing BCG plus durvalumab in BCG‐naive patients is currently ongoing. Another promising Phase II trial (NCT03759496)^85^ studied the efficacy and safety of durvalumab in patients with BCG‐refractory NMIBC. The maximum tolerated dose (MTD) and 1‐year HG relapse‐free rate of durvalumab are the primary end results.

Durvalumab showed a tolerable safety profile in combination with vicineum in a Phase I trial (NCT03258593)^86^ with a 12‐month CR rate of 17% in patients with BCG‐unresponsive HR‐NMIBC. It is also being evaluated in a Phase I/II RIDEAU study (NCT05120622)^87^ that aims to determine whether the use of systemic durvalumab with anti‐CTLA‐4 tremelimumab is effective in HR‐NMIBC patients. Finally, a Phase I/II trial DURANCE (NCT04106115)^88^ will evaluate durvalumab plus S‐488210/S‐488211, a 5‐peptide vaccine that stimulates a cytotoxic T‐lymphocyte response against tumor cells and induces tumor cell lysis.

#### 3.1.5 Sasanlimab

Sasanlimab is an anti‐PD‐1 inhibitor with antineoplastic activities that play an important role in tumor evasion from host immunity.^89^ It is currently being investigated in a randomized, Phase III clinical trial (NCT04165317)^89^ to evaluate the outcome of sasanlimab and alternative BCG regimens in the treatment of HR‐NMIBC. Cohort A consists of three study arms (A, B, and C) of BCG‐naïve participants. Both arms A and B, which consist of BCG plus Sasanlimab, will be compared to arm C, which involves the induction and maintenance of BCG alone. Cohort B will include BCG‐unresponsive patients with CIS (B1) or papillary disease (B2). The primary endpoints are EFS and CR rates. The study is estimated to be completed in 2026.

## 4 OTHER IMMUNOMODULATORS

Immune checkpoint inhibitors have dominated the field of novel treatments for NMIBC for the past few decades. Nevertheless, the advances in understanding BC biology have led to the discovery of many other promising targets. Moreover, several immunomodulators, such as nadofaragene firadenovec‐vncg, ALT‐803, and CG0070, have shown promising efficacy and are being evaluated in addition to several targeted immunotherapies in several clinical trials (Table 2).

**FIGURE 2:**
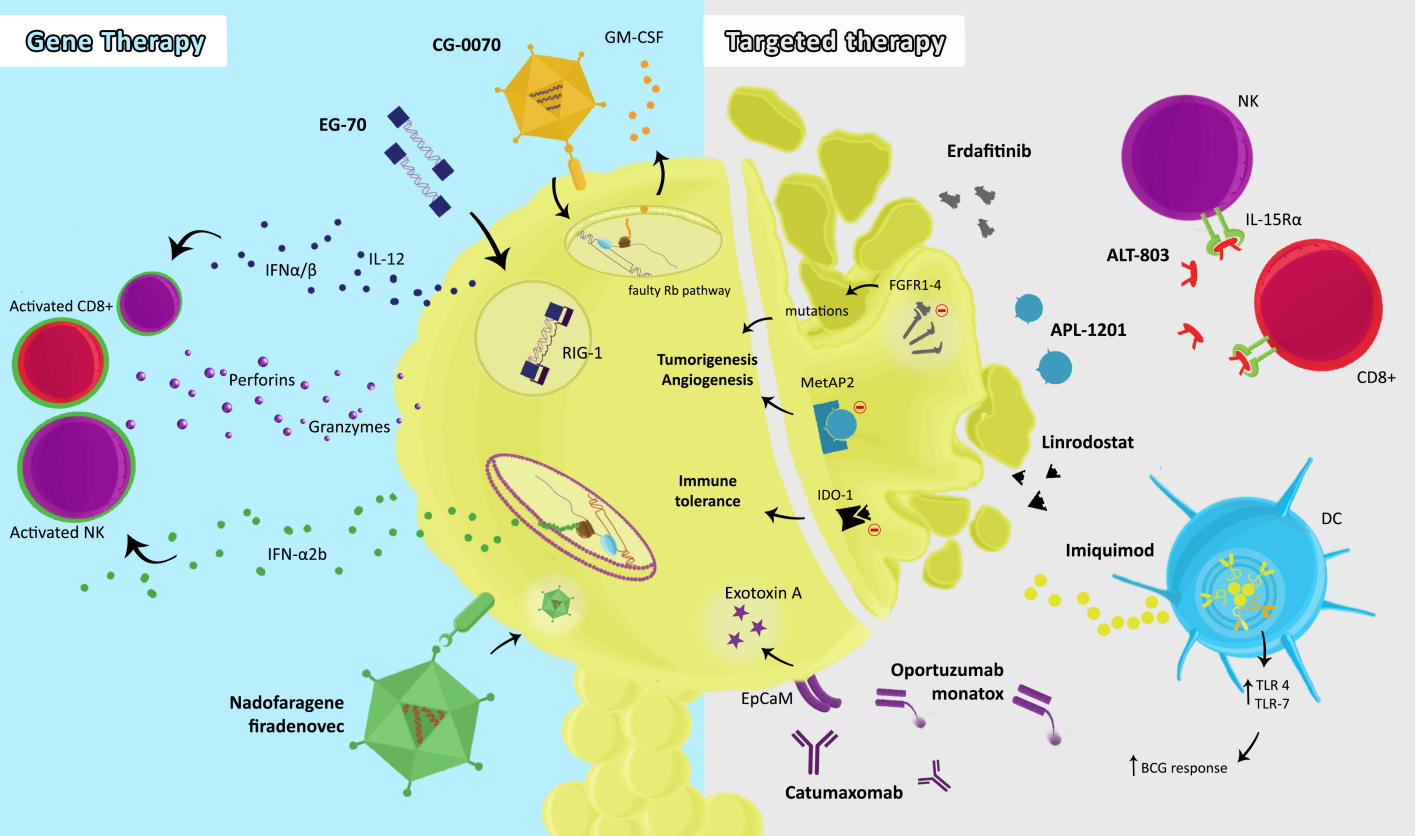
Alternative therapeutic options in high‐risk non‐muscle‐invasive bladder cancer. Nadofaragene firadenovec‐vcng and CG0070 are adenoviral carriers of cDNA encoding IFN‐α2b^96^ and GM‐CSF,^105^ respectively. CG0070 exploits tumoral cell faulty retinoblastoma pathway.^105^ EG‐70 binds to retinoic acid‐inducible gene‐1 (RIG‐1) to produce IFNα/β and increases levels of IL‐12.^118^, ^119^ Both interferons and IL‐12 activate CD8^+^ T‐cells and natural killers (NK), resulting in release of cytotoxic perforins and granzymes.^92^, ^94^ Catumaxomab and oportuzumab monatox target EpCaM, releasing exotoxin A.^151^, ^152^ Imiquimod acts as TLR‐7 agonist that reduces tumorigenesis and boosts BCG effects through upregulation of TLR‐4 and 7.^142^ Mutations in fibroblast growth factor receptor (FGFR) kinase and methionine aminopeptidases 2 (MetAP2) promote tumorigenesis.^155^, ^156^, ^160^ Whereas, increased indoleamine‐2,3‐dioxygenase 1 (IDO‐1) enzyme expression enhances immune tolerance.^164^ Linrodostat and APL‐1201 are inhibitors of IDO‐1^167^ and MetAP2^161^ receptors, respectively. Erdafitinib inhibits FGFR kinase,^156^ and ALT‐803 binds to IL‐15 receptor α subunit, releasing IL‐15 which acts on CD8^+^ T‐cells and NKs.^104^ BCG, Bacillus Calmette–Guérin; EpCaM, epithelial cell adhesion molecule; FGFR, fibroblast growth factor receptor; GM‐CSF, granulocyte‐macrophage colony stimulating factor; IDO‐1, indoleamine‐2,3‐dioxygenase 1; IFN, interferon; IL, interleukin; MetAP2, methionine aminopeptidases 2; NK, natural killer; RIG‐1, retinoic acid‐inducible gene‐1; TLR, toll‐like receptor.

Cytokines, such as interferon (IFN), interleukin 2 (IL‐2), and interleukin 15 (IL‐15), have been extensively studied in clinical settings of other cancers. Type I IFNs (IFN‐α and IFN‐β) are options that can be clinically utilized in the treatment of certain cancers, including melanomas, hematologic malignancies, Kaposi's sarcoma, and advanced renal carcinoma.^90^ Type 1 IFNs work on the JAK–STAT signaling pathway, leading to the secretion of IL‐4.^91^ They participate in the activation of cytotoxic T lymphocytes, macrophages, and natural killer cells.^92^ Additionally, they possess anti‐apoptotic and antiangiogenic effects on tumor cells and vasculature.^93^ IL‐15 is a part of a group of cytokines responsible for T‐cell (CD4^+^ and CD8^+^) proliferation and activation.^94^ IL‐15 also plays a role in the persistence of CD8^+^ memory T‐cells, which could contribute to antitumor long‐term immunity.^95^ Nadofaragene firadenovec‐vcng, CG0070, EG‐70, and BC‐819 are gene therapies that exploit cytokine mechanisms (Figure 2) and are being evaluated in NMIBC (Table 2).

### 4.1 Gene therapy

#### 4.1.1 Nadofaragene firadenovec‐vncg

Nadofaragene firadenovec‐vncg (rAd‐IFNa/Syn3) is a non‐replicating recombinant adenovirus vector that carries human recombinant IFN‐α2b cDNA with Syn3 formulation to enhance incursion into the bladder epithelium.^96^, ^97^ During a Phase I trial of the drug, intravesical nadofaragene firadenovec‐vncg established good tolerability without DLT.^98^ In another Phase II trial (NCT01687244), 14 patients (35.0%; 90% CI, 22.6% to 49.2%) did not have HG recurrence at 12 months.^99^ rAd‐IFNa/Syn3 was recently FDA approved^50^ for BCG‐unresponsive HR‐NMIBC after being evaluated in Phase III, multicenter, clinical trial (NCT02773849) which demonstrated 53.4% CR (55 out of 103), the highest CR at 3 months among FDA approved drugs, in patients with CIS ± Ta/T1 tumors.^100^ Though, approximately half of responders 45.5% (25 out of 55) maintained this response at 12 months. CR was defined as urine cytology, cystoscopy, and a nonmandatory bladder biopsy that did not display signs of low or HG recurrence. This demonstrates how nadofaragene firadenovec‐vcng, similar to other candidates, is more likely to postpone progression rather than achieve long‐term disease control. An additional Japanese, Phase III trial (NCT05704244)^101^ is currently evaluating nadofaragene firadenovec‐vncg in patients with HG, BCG‐unresponsive NMIBC. Primary results are expected in December 2024.

#### 4.1.2 CG0070

The retinoblastoma (Rb) pathway plays a critical role in regulating cell proliferation by interacting with many growth‐stimulating and inhibitory signals.^102^ Rb pathway defects are found in most cancers, including BC.^103^ Mutant Rb protein in bladder tumors lead to accelerated proliferation and invasion due to suppressed p53 and caspase‐3 signaling pathways, which in turn inhibit apoptosis of tumoral cells.^104^ CG0070 is a replicating oncolytic adenovirus that specifically attacks bladder tumor cells by exploiting their faulty Rb pathway through an E2F‐1 promoter.^105^ CG0070 also carries cDNA encoding granulocyte‐macrophage colony stimulating factor (GM‐CSF), a cytokine that has been previously described to promote antitumor activity in animal^105^, ^106^ and human models.^107^, ^108^

CG0070 yielded 47% CR (32%–62%) at 6 months in a Phase ll trial (NCT02365818)^109^ of all 45 BCG‐unresponsive patients. None with pure T1 disease had a 6‐month CR. However, CG0070 had a particular 58% CR (37%–78%) for patients with pure CIS and further demonstrated limited tumor progression in this category of patients.^110^ As a result, CIS may be the subset with the most favorable response to CG0070. Currently, a Phase III trial (NCT04452591)^111^ is being conducted on BCG‐unresponsive patients with CIS ± Ta/T1 to test CG0070 in this subset of patients. Results are expected in July, 2025. Additionally, CG0070 is currently being studied in conjunction with pembrolizumab in an open‐label, Phase II clinical trial (NCT04387461).^112^

In spite of several studies demonstrating the antitumor activity of GM‐CSF, numerous findings have shown otherwise. After exposure to GM‐CSF, multiple cancer cell types, including skin, lung, head and neck carcinoma, and neural gliomas, undergo stimulated cell proliferation and possibly metastasis through an immune‐independent mechanism.^113^, ^114^, ^115^, ^116^ GM‐CSF secretion and receptors have been identified and associated with the leukemoid reaction seen in advanced urothelial cancer.^117^ This raises questions on the GM‐CSF mechanisms in tumor progression, and further studies should explore the relationship between GM‐CSF and tumor progression, especially on the background of CG0070 treatment.

#### 4.1.3 EG‐70

EG‐70 is a novel nonviral nanoparticle combination of plasmids (nucleic acids) encoding for IL‐12, a pro‐inflammatory cytokine, and activators of the innate immune receptor retinoic acid‐inducible gene‐1 (RIG‐1).^118^ This type of gene therapy induces antitumor activity by employing a type‐1 IFN response through RIG‐1 receptor activation^119^ and coordinating adaptive and innate immune systems through IL‐12 release (Figure 2).^118^ A non‐randomized Phase l/ll trial (NCT04752722)^120^ of EG‐70 has shown good safety, tolerability, and therapeutic profile of intravesical EG‐70 in patients with BCG‐unresponsive NMIBC.^118^ CR was defined as the absence of HG recurrence through urine cytology, cystoscopy, and bladder biopsy, and 67% out of 14 patients achieved CR at week 12.^118^, ^120^ A Phase lll clinical trial is required to establish EG‐70's promising therapeutic effects.

#### 4.1.4 BC‐819

BC‐819 is a double stranded DNA plasmid which carries the A subunit of diphtheria toxin (dtA) under the regulation of an H19 promoter sequence.^121^ Accordingly, dtA synthesis would be triggered in the presence of H19, which is highly active in many human cancers, including BC, and nearly undetectable in normal tissue.^122^ BC‐819 can selectively impede protein synthesis in tumor cells causing cell death, which makes it a possible therapeutic option for NMIBC‐expressing H19. The safety and preliminary efficacy of intravesical BC‐189 was evaluated in a Phase l/ll trial of patients with BCG‐unresponsive disease.^123^ Tumor marker ablation occurred in 22% (4 out of 18) of patients. DFS greater than 35 weeks occurred in 5 out of 9 patients who received monthly maintenance therapy. No DLTs were observed. Further studies need to substantiate the efficacy of BC‐819 in this subset of patients.

### 4.2 Other targets

In addition to oncolytic viral therapy, including nadofaragene firadenovec‐vcng, CG0070, and novel gene therapies like EG‐70 and BC‐819, additional targeted therapies are being evaluated in several clinical trials (Table 2) as monotherapies or combinational regimens.

#### 4.2.1 IL‐15Rα

ALT‐803, also called N‐803, is a mutant IL‐15 superagonist fusion protein complex. By binding to IL‐15Rα, ALT‐803 enhances the effects of CD8^+^ T‐cells and NK through increased expression of granzyme B and perforins (Figure 2).^124^ A Phase l/llb trial, QUILT‐2.005 (NCT02138734),^125^ of combined ALT‐803 and BCG achieved a 100% CR rate (*n* = 7) at 24 months without disease recurrence, which prompted the initiation of a Phase ll/lll trial with the same intervention. Results of the Phase II/III trial QUILT‐3.032,^126^ which included BCG‐unresponsive NMIBC patients demonstrated a CR rate at any time of 71% in patients with CIS (*n* = 59/83) which is much higher than that of pembrolizumab (41%)^70^ and that of nadofaragene (53%).^100^ 59% of patients maintained CR for at least 12 months, and the median duration of response among responders was 24.1 months. Among patients with papillary disease, 57% achieved a DFS rate at 12 months and 48% at 24 months.^126^, ^127^ Of all completed trials of BCG‐unresponsive disease, only ALT‐803 surpassed the proposed clinically relevant thresholds.^128^ These results are promising but require further validation in larger prospective cohorts.

#### 4.2.2 TLR

Toll‐like receptors (TLRs) are pattern recognition receptors (PRRs) that are predominantly involved in innate immune responses by recognizing various pathogen‐associated molecular patterns (PAMPs).^129^ Activation of TLRs can lead to both inhibited and stimulated tumorigenesis through different mechanisms. Expression of TLR on tumoral cells activates the NF‐kB signaling pathway, promoting the production of various cytokines and chemokines that play an integral role in tumor proliferation,^130^ immune tolerance,^130^ and angiogenesis^131^ in the TME. On the other hand, TLRs, mainly TLR4 and TLR3, expressed on dendritic cells (DC) and other antigen‐presenting cells (APC) play a role in DC activation,^132^ maturation,^133^ and migration.^134^ Mature DCs are able to induce antitumor immune responses through T‐helper 1 (Th1)‐induced cytotoxicity.^130^ Factors found in the TME, such as COX‐2, PGE_2_, IL‐6, IL‐1, VEGF, and TGF‐β, suppress maturation of myeloid DCs,^130^ diminishing tumor antigen‐specific immunity.

Compared to normal bladder urothelium, NMIBC expresses a moderate proportion of TLRs 2, 3, 4, 5, 7, and 9.^135^ Th1‐mediated cytotoxicity of TLR agonists against bladder carcinoma cell lines has been previously reported in vivo.^136^ Successful intravesical therapy with BCG in a significant proportion of patients suggests that other TLR agonists are worthy of exploration. For instance, unlike BCG, which is My98‐dependent on activation of TLR 2 and 4, P‐MAPA (Protein Aggregate Magnesium‐Ammonium Phospholinoleate‐Palmitoleate Anhydride) is a novel treatment that also targets TLR 2 and 4 but leads to a distinct increase in interferon signaling pathway (TRIF‐dependent pathway), which was more effective in the treatment of animal NMIBC models.^137^

Given the moderate antitumor responses of TLR agonists, accumulating preclinical evidence supports synergistic use,^138^, ^139^, ^140^ rather than monotherapy, of TLR agonists.^141^ Imiquimod (TLR7 agonist) in combination with BCG was investigated in animal models with NMIBC and was shown to reduce tumorigenesis and boost BCG effects through up‐regulation of TLR4 and TLR7 and downregulation of P70S6K1 protein.^142^

The first human clinical trial (NCT05710848)^143^ to determine the safety and tolerability of STM‐416, a TLR7 and TLR8 agonist, in patients with high‐risk recurrent NMIBC after standard of care treatment is currently ongoing. Additionally, TARA‐002 is a non‐virulent genetically edited strain of Streptococcus pyogenes that targets TLR4. It will be evaluated in a recruiting, Phase la/b clinical trial ADVANCED‐1 (NCT05085990),^144^ which will be followed up by Phase II trial ADVANCED‐2 (NCT05951179).^145^ However, the study will involve three cohorts that do not involve BCG‐unresponsive disease: BCG‐naive, BCG‐intolerant, and BCG‐recurrent or persistent.

#### 4.2.3 EpCaM

The epithelial cell adhesion molecule (EpCaM), which is involved in upregulating c‐myc and cyclins A/B,^146^ is expressed in many tumor tissues, including urothelial carcinoma of the bladder.^147^, ^148^ On the other hand, expression of EpCAM is weak and restricted to superficial cells in normal urothelium.^149^ EpCAM can be a potential target for immunotherapy due to its strong association with HG, advanced‐stage, and poor OS in bladder carcinoma.^150^ Catumaxomab, a bispecific EpCaM monoclonal antibody, has been tested for the first time in two patients with BCG‐recurrent NMIBC.^151^ With a good safety and tolerability profile, recurrence‐free intervals were 32 and 35 months. Oportuzumab monatox is a recombinant fusion protein including an EpCaM fragment antibody fused to a variant of Pseudomonas exotoxin that functions by blocking protein synthesis and promoting an adaptive T‐cell‐mediated antitumor response.^152^ Oportuzumab monatox demonstrated a CR of 39% (29%–49%, 95% CI) (*n* = 93) at 3 months in a Phase lll clinical trial (NCT02449239).^153^ Among responders, 52% remained free of CIS 12 months after treatment initiation.^152^ Therefore, oportuzumab monatox or catumaxomab might represent a promising immunotherapeutic approach for the treatment of BCG‐unresponsive NMIBC (Figure 2).

#### 4.2.4 FGFR

The fibroblast growth factor receptors (FGFR), which are a family of receptor tyrosine kinases found on the cell membrane, play an important role in the development, proliferation, and overall hemostasis of cells.^154^ FGFRs respond to extracellular stimulation by activating cellular growth, differentiation, and migration.^154^ As a result, mutations in FGFR have been linked to various pathologies and syndromes related to tumorigenesis, especially NMIBC, where active mutations in FGFR3 have been found in (20%–80%) of patients, depending on tumor stage.^155^, ^156^ FGFR1‐4 inhibitor erdafitinib has FDA approval for the treatment of recurrent metastatic BC.^157^ Its efficacy and safety are currently being evaluated compared to gemcitabine or mitomycin‐c in a Phase II trial (NCT04172675)^39^ that included BCG‐recurrent NMIBC patients with somatic FGFR3 mutations or fusions. Another Phase II trial (NCT04917809)^158^ is testing whether oral erdafitinib is effective in treating recurrent NMIBC patients with oncogenic FGFR3 mutations after BCG or chemotherapy. The primary outcome measured is CR through urine cytology, cystoscopy, and biopsy. The trial is estimated to be complete in August 2023. Notably, dovitinib, an oral receptor tyrosine kinase (RTK) inhibitor that possesses inhibitory effects on FGFR3 and vascular endothelial growth factor receptor RTKs, demonstrated failure in a Phase II trial to treat patients with BCG‐refractory NMIBC with FGFR3 due to frequent treatment‐related toxicities.^159^ Dose reductions were required in 77% of patients enrolled. The 6‐month CR rate was 8%, and the primary endpoint was not achieved.^159^

#### 4.2.5 MetAP2

Methionine aminopeptidases 2 (MetAP2) are a class of metalloproteases that function by removing the N‐terminal methionine from newly synthesized proteins. MetAP2 demonstrated a crucial positive effect on tumorigenesis and angiogenesis.^160^ Therefore, MetAP2 inhibition has been of interest for its antiangiogenic effect and tumor growth inhibition.

APL1202 is a reversible and orally available selective MetAP2 inhibitor that was evaluated in a Phase 2 trial (NCT04498702).^161^ Among BCG‐unresponsive NMIBC patients, 54.3% (95% CI 37.2%–73.2%) was the one‐year recurrence‐free rate.^161^

APL1202 is also being evaluated in a Phase III trial (NCT04736394)^162^ in BCG‐naïve, Intermediate‐risk NMIBC patients, which is expected to be complete in 2025. Moreover, an ongoing Phase III trial (NCT04490993)^163^ is being conducted to evaluate the clinical safety and efficacy of APL‐1202 in combination with epirubicin hydrochloride in intermediate and chemo‐refractory HR‐NMIBC patients.

#### 4.2.6 IDO1

Indoleamine‐2,3‐dioxygenase 1 (IDO1) is an enzyme that plays an important immunoregulatory role through tryptophan metabolism.^164^ The overexpression of IDO1 in tumor cells and multiple immune cells in the tumor microenvironment induces T‐cell anergy and thus promotes immune tolerance.^164^ BMS‐986205 (Linrodostat) is an IDO1 inhibitor that is yet to be evaluated as monotherapy in the setting of NMIBC. The blockage of IDO1 may be used clinically to inhibit tumor evasion from immune surveillance.

A small number of patients may develop ICI resistance through TME suppression.^165^, ^166^ As a result, the combination of IDO‐1 inhibitors, which can overcome immune suppression, and anti‐PD‐1, which promote antitumor activity, may enhance T‐cell‐mediated antitumor responses. Interleukin‐2 (IL‐2) production and CD8^+^ T cells are immediately restored within the TME by doublets of CTLA‐4 or PD‐1/PD‐L1 with IDO blockage.^167^ Check‐Mate 9UT (NCT03519256), a Phase ll clinical trial in patients with high‐risk BCG‐unresponsive NMIBC, evaluated BMS‐986205 plus nivolumab.^168^ The trial was terminated due to insufficient accrual. Thus, the role of anti‐IDO treatments as monotherapy or in combination with ICIs for NMIBC has yet to be thoroughly studied.

Overall, IL‐15Rα, TLR, MetAP2, EpCaM, FGFR, and IDO1 present promising targets (Figure 2) that are being evaluated in clinical trials involving BCG‐unresponsive HR‐NMIBC (Table 2).

## 5 COMBINATION THERAPY

The implication of combination therapy has proven to be beneficial in various clinical trials in the setting of BCG‐naïve or BCG‐unresponsive NMIBC. ICIs have shown synergistic effects with various other immunomodulators, chemotherapeutic agents, and BCG. Monotherapy with immunomodulators alone has not shown long‐term disease control. Thus, many combination strategies are currently under evaluation.

Myeloid‐derived suppressor cells (MDSC) can prompt immune tolerance and impede the recognition of BC cells by immune cells.^169^ Thus, depleting MDSCs, which is an effect shown by cisplatin, can maintain antitumor function of CD8^+^ T cells.^169^ The combination of ICI and chemotherapeutic agents has been extensively tested in advanced BC, since chemotherapy is mostly indicated in this patient population. Furthermore, studies have indicated that gemcitabine may harbor immune modulating properties on the TME.^170^ Clinical trials involving gemcitabine and ICIs for NMIBC are underway. In the setting of BCG‐unresponsive HR‐NMIBC, pembrolizumab is being evaluated with gemcitabine in an ongoing Phase II trial (NCT04164082).^171^ SunRISe‐1 (NCT04640623),^172^ a Phase IIb study, and SunRISe‐3 (NCT05714202),^173^ a Phase III trial, are testing systemic cetrelimab (anti‐PD‐1) with TAR‐200, an intravesical drug delivery system of gemcitabine, as combination and monotherapies. Another Phase II study (NCT05418309)^174^ of nab‐paclitaxel in combination with tislelizumab (anti‐PD‐1) for HR‐NMIBC that cannot be completely resected is currently ongoing. In a randomized, clinical trial (KEYNOTE‐361) of patients with advanced urothelial carcinoma, the addition of pembrolizumab to first‐line platinum‐based chemotherapy proved insignificant.^175^ The efficacy of combination therapy between ICI and chemotherapeutic agents is yet to be determined in HR‐NMIBC.

The blockage of CTLA‐4 is thought to enhance T‐cell proliferation early in an immune response, primarily in lymph nodes, whereas anti‐PD‐1 enhances antitumor T cells primarily in peripheral tissues.^176^, ^177^ However, the increased rate of significant IRAEs in ICI combinations represents a challenge regarding the integration of such treatments if they are proven effective. The combination of multiple ICIs could provide a synergistic immune response against BC cells. The combination of anti‐CTLA‐4 agent tremelimumab and anti‐PD‐L1 agent durvalumab will be evaluated in NCT03317158 and NCT05120622.^83^, ^87^

Targeted therapies are also viable options for combination. IDO peptide‐based immune‐modulatory therapeutic (IO102‐IO103) is a novel investigational candidate that can enhance T‐cell‐mediated antitumor responses when combined with PD‐L1 inhibition,^178^ Moreover, the combination of IDO1 inhibitor, BMS‐986205, and ICI has not been evaluated yet since the only trial did not meet sufficient patient enrollment.^168^ FGFR inhibitors, such as vofatamab and rogaratinib, are other candidates that have shown promising results in metastatic urothelial carcinoma when combined with atezolizumab or pembrolizumab.^179^, ^180^ However, clinical trials evaluating FGFR inhibitors in combination with ICI in HR‐NMIBC are lacking. Moreover, oportuzumab monatox, which utilizes EpCaM, is currently being evaluated in a Phase I study (NCT03258593)^86^ in combination with durvalumab.

Many ICIs were found to be synergistic with BCG therapy. As mentioned earlier, BCG‐resistant patients have enhanced PD‐L1 expression on tumoral tissue, which makes the combination a promising option.^68^ Studies including dual ICI and BCG therapy have been mentioned earlier and are outlined in Table 1. Moreover, BCG has been evaluated with Interferon alpha (IFN‐α) in a Cochrane review, and no significant differences in recurrence or progression were present when comparing BCG plus IFN‐α and BCG alone in the treatment of NMIBC.^181^ BCG was also studied in combination with the receptor tyrosine‐kinase inhibitor Sunitinib in a Phase II trial (NCT00794950)^182^ where a 3‐month CR of 77% (95% CI [55, 86]) was demonstrated. 2‐year RFS was 77% (95% CI [58, 88]) and 2‐year PFS was 100%. However, AEs were common (34/36 patients), but only a few AEs (4.5% [6/133]) were ≥ grade 3.^182^ Finally, BCG combination therapy with ALT‐803,^126^, ^127^ as discussed earlier, was shown to be the most promising.

## 6 FUTURE PERSPECTIVES

FDA‐approved drugs for BCG‐unresponsive CIS ± Ta/T1 NMIBC, valrubicin, pembrolizumab, and, most recently, nadofaragene firadenovec‐vcng, have not achieved the clinically relevant outcomes of 6‐month 50% and 12‐month 30% CRR that were previously proposed by the FDA, IBCG, and American Urological Association.^27^, ^128^, ^183^ Studies involving other novel ICIs are underway. The efficacy and safety of other ICIs including HX008, tislelizumab, and camrelizumab are currently under evaluation in multiple clinical trials. HX008 (pucotenlimab) is a novel humanized anti‐PD‐1 monoclonal antibody which is being evaluated in a Phase II clinical trial for BCG‐unresponsive NMIBC.^184^ Results are expected in December 2023. Intravenous and intravesical camrelizumab, an anti‐PD‐1, are being compared in the BCG‐unresponsive setting in a Phase I/II randomized study.^185^ Tislelizumab is only being evaluated in HR‐NMIBC as combinational therapy. TACBIN‐01^186^ and SPARE‐007^187^ are examining the safety and efficacy of BCG combined with Tislelizumab in a BCG‐naive setting. Tislelizumab is also being evaluated in combination with nab‐paclitaxel and in combination with disitamab vedotin, a human epidermal growth factor receptor 2 (HER2) antibody conjugate, in TRUCE‐22,^174^ and TRUCE‐04,^188^ respectively. These studies are preliminary, and further trials need to investigate the therapeutic efficacy of these monotherapies and combinational therapies.

PD‐1 and PD‐L1 are the predominant immune checkpoints involved in clinical trials of HR‐NMIBC. There is a shortage of trials including CTLA‐4 and other immune checkpoints. Lymphocyte‐activation gene 3 (LAG‐3) (CD223) is a negative immune checkpoint that plays an important role in T‐cell‐regulatory‐mediated suppression of immune response to cancer upon binding to its ligand, major histocompatibility complex class II (MHC II).^189^ T‐cell immunoglobulin and immunoreceptor tyrosine‐based inhibitory motif domain (TIGIT) is another emerging inhibitory immune checkpoint that is mostly expressed on memory T cells, natural killer (NK) cells, and T‐regulatory (Treg) cells.^190^ Through binding to its ligands, CD155 and CD112, TIGIT directly inhibits T‐cell activity and NK‐cell cytotoxicity in the tumor microenvironment (TME), potentiating immune evasion.^191^ Single cell sequencing on bladder tumor cells revealed high expression of TIGIT on Treg cells.^192^ TIGIT has also been associated with worse recurrence‐free outcomes.^193^ Targeting TIGIT reversed immunosuppression and inhibited IL‐32, which plays a role in BC metastasis.^192^ LAG‐3, PD‐1, and TIGIT are all co‐expressed in tumor‐infiltrating lymphocytes; thus, dual LAG‐3/PD‐1 or TIGIT/PD‐1 blockage may synergistically bypass immune resistance and potentiate treatment efficacy.^192^, ^194^ LAG‐3 and TIGIT can become the primary targets next to PD‐1 in the development of cancer therapy. However, clinical evidence on the use of anti‐LAG‐3 or anti‐TIGIT is lacking and is being explored in cohort C of KEYNOTE‐057.^195^

Gene therapy is an option with promising potential in HR‐NMIBC. There is an increased interest in oncolytic viral therapy, especially after the FDA‐approval of nadofaragene firadenovec‐vcng. CG0070, EG‐70, and BC‐819 are other gene‐delivery agents under evaluation. Despite some promising results, further research is required to optimize the safety and efficacy of gene therapy in BCG‐unresponsive HR‐NMIBC.

ICIs synergistic capability with other combinational immunomodulators, chemotherapy, and BCG has been discussed earlier. Recently, many molecular targets are emerging as candidates for BC therapy. Many of these novel treatments are currently under investigation for HR‐NMIBC. Targets including IL‐15Rα, TLR, EpCaM, FGFR, MetAP2, and IDO1 are options that may be exploited for combinational of monotherapy treatment strategies. Given the background of IL‐15 promising advances in cancer immunotherapy, another IL‐15 superagonist, SHR‐1501, is being currently studied as monotherapy or in combination with BCG in a Phase l/ll study.^196^ Enfortumab vedotin (EV) is an antibody drug conjugate directed against Nectin‐4, a cell adhesion molecule overexpressed in urothelial carcinoma.^197^ EV has shown significant prolonged survival compared to chemotherapy in metastatic BC.^198^ Safety and tolerability EV in patients with BCG‐unresponsive HR‐NMIBC is currently being evaluated in a Phase I trial EV‐104 (NCT05014139).^199^ Furthermore, mycobacterium phlei cell wall‐nucleic acid complex (MCNA) is a novel therapeutic compound that exerts its effects through an indirect immunomodulatory effect by stimulating the synthesis of multiple cytokines, including IL‐6, IL‐8, IL‐12, IL‐18 and TNF‐ α. It also acts as a direct chemotherapeutic agent though tumor‐selective apoptosis.^200^ MCNA has been evaluated in a Phase II/III trial (NCT00406068)^201^ that involved 129 patients with BCG‐refractory disease. Overall DFS was 25% and 19% at 1 and 2 years, respectively. DFS was notably higher in patients with papillary only tumors in comparison to CIS alone or with Ta/T1.^202^ Further research needs to investigate novel molecular targets that can enhance treatment development.

According to Rose et al.,^128^ there is a lack of uniformity in clinical trials including BCG‐unresponsive NMIBC. This further diminishes the reliability of outcomes observed in the meta‐analysis of these agents. Furthermore, the lack of consensus on a standardized control treatment is an additional challenge that must be addressed to upgrade the reliability of clinical trials. Single‐arm controlled trials are considered suitable by the FDA for this subset of patients when RCTs are unethical or unfeasible due to the unavailability of an effective bladder‐sparing treatment.^24^ This led to the approval of treatments with limited efficacies. However, Tan et al.^203^ consider randomization to be an achievable approach in the present day that can be required for the evaluation of therapeutic options before regulatory approval. As a result, RCTs with an ideal control treatment are required to eliminate risks of bias and increase the reliability of meta‐analyses.^128^ Furthermore, tumor characterization and subtype identification is becoming imperative for enriching our understanding of treatment response and disease biology. The 2022 edition of the World Health Organization classification of tumors of the urinary tract underscores the need for reporting the presence and percentages of different subtypes in urothelial carcinomas.^8^ The development of clinical trials with adequate patient and tumor characterization, subtype classification, and choice of end points is critical to maintain uniformity across the literature.

## 7 CONCLUSION

Patients with BCG‐unresponsive HR‐NMIBC have very limited therapeutic options, and the life‐altering option of RC might be a necessity for optimal disease control. The development of bladder‐preserving therapies for BCG‐unresponsive HR‐NMIBC represents a challenge in the modern era. Despite the most recent FDA approval of nadofaragene firadenovec‐vncg and the availability of pembrolizumab and valrubicin, there is a significant unmet need for less expensive, bladder‐preserving therapies that can offer long‐term disease control, significantly improve quality of life, and potentially replace BCG. Immune checkpoint inhibitors, including anti‐PD‐1 and anti‐PD‐L1 agents, are being extensively studied and have shown promising results with the approval of pembrolizumab. However, there is a lack of trials including CTLA‐4, LAG‐3, and TIGIT immune checkpoints. Other targets, including IL‐15Rα, TLR, EpCaM, FGFR, IDO1, and MetAP2, may be exploited for combinational treatment strategies. Gene‐delivery agents, including CG0070, EG‐70, and BC‐819, are another promising options that still require further evaluation. Further research needs to address the landscape of biomechanisms in HR‐NMIBC to discover targets that are essential for treatment development. Maintaining uniformity across clinical trials is likewise fundamental for yielding reliable outcomes.

## AUTHOR CONTRIBUTIONS

**Zein Alabdin Hannouneh:** Investigation (equal); project administration (lead); writing – original draft (lead); writing – review and editing (lead). **Amjad Hijazi:** Investigation (equal); writing – original draft (equal); writing – review and editing (equal). **Alaa Aldeen Alsaleem:** Investigation (equal); writing – original draft (supporting); writing – review and editing (equal). **Siwan Hami:** Investigation (equal); writing – original draft (supporting); writing – review and editing (equal). **Nina Kheyrbek:** Investigation (supporting); writing – original draft (equal). **Fadi Tanous:** Investigation (supporting); writing – original draft (supporting). **Karam Khaddour:** Conceptualization (lead); supervision (equal); writing – review and editing (lead). **Abdulfattah Abbas:** Supervision (equal); writing – review and editing (supporting). **Zuheir Alshehabi:** Project administration (lead); supervision (lead); writing – review and editing (supporting).

## CONFLICT OF INTEREST STATEMENT

The authors declare no conflict of interest.

## Data Availability

Data sharing is not applicable to this article as no new data were created or analyzed in this study.
